# Cloud Service Selection Using Multicriteria Decision Analysis

**DOI:** 10.1155/2014/459375

**Published:** 2014-02-13

**Authors:** Md Whaiduzzaman, Abdullah Gani, Nor Badrul Anuar, Muhammad Shiraz, Mohammad Nazmul Haque, Israat Tanzeena Haque

**Affiliations:** ^1^Faculty of Computer Science & Information Technology, University of Malaya, 50603 Kuala Lumpur, Malaysia; ^2^School of Electrical Engineering and Computer Science, The University of Newcastle, Callaghan, NSW 2308, Australia; ^3^Department of Computing Science, University of Alberta, Edmonton, AB, Canada P6G 2M7

## Abstract

Cloud computing (CC) has recently been receiving tremendous attention from the IT industry and academic researchers. CC leverages its unique services to cloud customers in a pay-as-you-go, anytime, anywhere manner. Cloud services provide dynamically scalable services through the Internet on demand. Therefore, service provisioning plays a key role in CC. The cloud customer must be able to select appropriate services according to his or her needs. Several approaches have been proposed to solve the service selection problem, including multicriteria decision analysis (MCDA). MCDA enables the user to choose from among a number of available choices. In this paper, we analyze the application of MCDA to service selection in CC. We identify and synthesize several MCDA techniques and provide a comprehensive analysis of this technology for general readers. In addition, we present a taxonomy derived from a survey of the current literature. Finally, we highlight several state-of-the-art practical aspects of MCDA implementation in cloud computing service selection. The contributions of this study are four-fold: (a) focusing on the state-of-the-art MCDA techniques, (b) highlighting the comparative analysis and suitability of several MCDA methods, (c) presenting a taxonomy through extensive literature review, and (d) analyzing and summarizing the cloud computing service selections in different scenarios.

## 1. Introduction

Cloud computing (CC) is the distributed computing model which provides computing facilities and resources to the users in an on-demand pay-as-you-go model [1]. The aim of the cloud computing model is to increase the opportunities for cloud user by accessing leased infrastructure and software applications from anywhere anytime manner. Therefore, cloud computing offers a new kind of information and services that widen the new vision of information technology (IT) services [2, 3]. The recent hype of the cloud computing and at the same time the rise of the smart mobile devices help us to envision mobile cloud computing (MCC). MCC is a distributed computing model comprise with cloud computing, mobile computing and seamless connectivity [4, 5]. The objective of MCC is to enhance the computing and processing power of the mobile devices by offloading tasks to cloud data center [6–10]. In the cloud, the resources are hosted as software, database services, virtual servers (virtual machines), hardware, complete service workflows, or complex configurations of distributed computing systems and applications for provisioning [11, 12]. These resources are provisioned as services and offered to the customer by the cloud service provider (CSP) [13].

Service provisioning is an important aspect of CC because it directly impacts the user experience of the service. New users and companies seeking cloud services are constantly emerging. The vast diversity among the available cloud services makes it difficult for the customer to decide whose services to use or even to determine a valid basis for their selection [14, 15]. Such decision problems have recently attracted considerable attention from industry and academia. Loyola and Franklin, for example, have explicitly applied multiple criteria decision analysis (MCDA) to assist with decision-making. MCDA is modeled after the way humans are thought to make decisions. Although a variety of different MCDA methods, techniques, and approaches have been studied, the basic ingredients of MCDA are the same: a finite or infinite set of actions, at least two criteria, and one decision maker (DM). With these elements, MCDA assists in decision making mainly by choosing, ranking, or sorting the actions. Consequently, MCDA is not only a collection of theories, methodologies, and techniques but also a specific perspective for dealing with decision-making problems [16].

Over the past few decades, decision-making theory has been successfully applied in a growing number of diverse domains and has assisted in decision making, including several well-known examples. The multicriteria decision-making (MCDM) approach is capable of handling multiple conflicting criteria [17]. This study provides an in-depth analysis of several MCDA/MCDM-related methods and models, as well as some insight into the state-of-the-art in decision analysis techniques [18, 19]. Multicriteria decision making is a subfield of operations research that addresses techniques to solve multicriteria problems such as the cloud service selection problem. All MCDA methods depend on a matrix called the evaluation matrix, decision matrix, and payoff matrix or evaluation table [20].

One objective of this study is to survey the literature and to provide a critical assessment of the available MCDA techniques and their usage in service selection for cloud computing. A literature review is used to demonstrate the integration of MCDA techniques and cloud computing based on their usage and popularity. Hence, in this paper, we reviewed the current literature and identified the different types of problems. The limitations of the various methods and techniques and their pros and cons are also discussed. We highlight the fact that most MCDA techniques have often been used individually in previous studies. Finally, we provide crucial information through reviewing the available literature on MCDA techniques and its usage in service selections in cloud computing [18].

Hence, we identify our contribution of this review are four-fold: (a) focusing on the state-of-the-art MCDA techniques, (b) highlighting the comparative analysis and suitability study of several MCDA methods, (c) presenting a taxonomy through extensive literature review, and (d) analyzing and summarizing the cloud computing service selections in different practical implementation aspects.

The rest of this paper is organized as follows.  Section 2 describes the background of MCDA.  Section 3 discusses the fact that several MCDA applied methods can be applied in MCDM.  Section 4 describes the performance of several of these methods as applied to cloud computing service selection.  Section 5 provides the conclusion. The list of acronyms frequently used in this paper is shown at the end of the paper.

## 2. Background of MCDA

**Figure 1:**
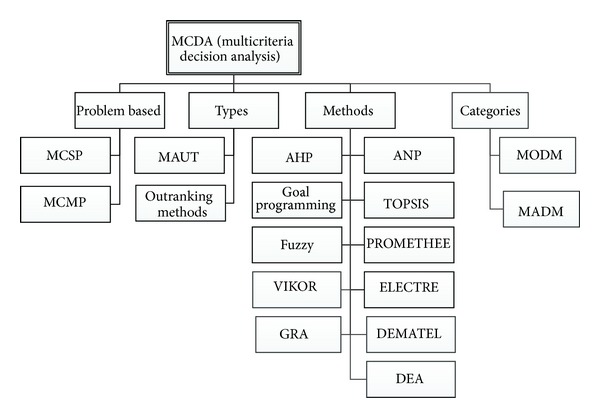
Taxonomy of MCDA.

In 1951, Harold William Kuhn and Albert William Tucker introduced the vector maximum problem, the first explicit consideration of the basic concepts of MCDA. In 1972, a conference on “Multiple Criteria Decision Making (MCDM)” was held at Columbia University in South Carolina. Since then MCDA/MCDM has experienced rapid growth and continues to grow today [16]. A taxonomy of MCDA is presented in Figure 1.

MCDA, one of the most important branches of operations research, aims to design mathematical and computational tools for selecting the best alternative among several choices, with respect to specific criteria, either by a single decision maker or by a group. The field consists of two main categories: multiattribute decision making (MADM) and multiobjective decision making (MODM). According to a survey conducted to evaluate the use of different methods, especially for improving quality, fuzzy was the most frequently used, representing 40% of the total, followed by AHP and ANP together at approximately 20%. TOPSIS was also widely used. In contrast, DEA, Goal Programming, and ELECTRE were employed only rarely [21].

- MCDM is a collection of methodologies for comparing, ranking, and selecting multiple alternatives, each having multiple attributes. It depends on a matrix called the evaluation matrix, decision matrix, payoff matrix, or evaluation table.
- MCSP selects the best alternative from a finite set of alternatives, all of which are known a priori.
- MCMP selects the best alternative from a very large or infinite set of alternatives, not all of which are known a priori.
- MAUT finds a utility function reflecting the usefulness of a particular alternative.

## 3. MCDA Methods

MCDA methods can be categorized into two types: (1) multiattribute utility theory (MAUT) and (2) outranking methods. MAUT attempts to find a function reflecting the utility or usefulness of a particular alternative. Each action is assigned a marginal utility, with a real number representing the preferability of the considered action. The returned utility is the sum of these marginal utilities. Outranking methods decide whether one alternative is ranked higher than another by employing a pairwise comparison.

MCDA methods are divided into multiobjective decision making (MODM) and multiattribute decision making (MADM). The two methods differ mainly by how the alternatives are enumerated. In MODM, they are not predetermined but arise from the optimization of a set of objective functions. In MADM, they are predetermined, and a small subset is evaluated against a set of attributes. In both methods, the best alternative is chosen by comparing the rankings of each alternative/attribute combination [16, 20].

### 3.1. Analytic Hierarchy Process (AHP)

In 1980, Thomas L. Saaty discovered AHP, a popular and widely used method for MCDA. AHP allows the use of qualitative as well as quantitative criteria when evaluating alternatives and the attributes are not entirely independent of each other. The AHP is based on a pairwise comparison, with the attributes structured into a hierarchal relationship, which is very useful. The hierarchy starts from the top level towards the goal; the lower levels correspond to criteria, subcriteria, and so on. In this hierarchy tree, the process starts from leaf nodes and progresses up to the top level. Each output level represents the hierarchy corresponding to the weight or influence of different branches originating for that level. Finally, after making the comparisons, the best alternative with respect to each attribute is usually selected [22].

### 3.2. Analytic Network Process (ANP)

ANP is an extension of AHP proposed by Thomas L. Saaty in 1996. It is a comprehensive decision-making technique designed to overcome the problem of dependence and feedback among the criteria, using unidirectional hierarchical relationships between decision levels. ANP describes interrelationships among the decision levels and attributes using unidirectional hierarchical relationships with dependence and feedback, employing ratio scale measurements based on pairwise comparisons to model the decision problem. To handle interdependence among elements, ANP derives a “supermatrix” containing composite weights [5]. ANP has been applied successfully in many real-world decision-making problems [23].

### 3.3. Technique for Order of Preferences by Similarity to Ideal Solution (TOPSIS)

This technique shows preference for the similarity to an ideal solution, which tries to select an alternative that is closest to the ideal solution and simultaneously farthest from the anti-ideal solution. In this technique, the decision matrix is first normalized using vector normalization, and the ideal and anti-ideal solutions are identified within the normalized decision matrix.

TOPSIS was developed in 1981 by Hwang and Yoon, and it selects alternatives having the shortest distance from the positive ideal solution and the farthest distance from the negative ideal solution [21]. It is a multiple criteria method for identifying solutions from a finite set of alternatives. The optimal solution should have the shortest distance from the positive ideal solution and the farthest from the negative ideal solution [22]. The TOPSIS method introduces an aggregating function, including the distances from the ideal point and from the negative-ideal point without considering their relative importance. However, the reference point could be a major concern in decision making, and it should be as close as possible to the ideal solution [20, 22].

### 3.4. Elimination and Choice Expressing Reality (ELECTRE)

ELECTRE was developed in 1991 by Roy and colleagues at the SEMA Consultancy Company. Several versions of this method have been developed since (ELECTRE I, ELECTRE II, ELECTRE III, ELECTRE IV, ELECTRE IS, and ELECTRE TRI (ELECTRE Tree)). ELECTRE consists of two sets of parameters: the importance coefficient and the veto thresholds [21].

This method falls in the class of outranking MCDM methods. In comparison with the previously discussed methods, this method is computationally intricate: the simplest variant of ELECTRE involves up to 10 steps. It performs a pairwise comparison between the alternatives in order to determine their outranking relationships. These relationships are then used to identify and eliminate alternatives that are dominated by others, yielding a smaller set of alternatives.

The ELECTRE method handles discrete criteria that are both quantitative and qualitative in nature, providing complete ordering of the alternatives. Alternatives are preferred over most of the criteria and depend on concordance, discordance indices, and threshold values and graphs of relationships. These graphs are used in an iterative procedure to obtain the ranking of alternatives [20, 22].

### 3.5. Preference Ranking Organization METHod of Enrichment Evaluations (PROMETHEE)

PROMETHEE was developed in the mid-1980s by Brans and Vincke. It is an improved form of the outranking method ELECTRE, but it differs from ELECTRE in the pairwise comparison stage. Whereas both PROMETHEE and ELECTRE determine whether one particular alternative is better than another, PROMETHEE additionally considers the degree to which it is better, using this piece of information to eliminate dominated alternatives and to identify nondominated or least dominated alternatives. PROMETHEE ranks the alternatives and is easier to use and less complex than ELECTRE [22].

### 3.6. Decision-Making Trial and Evaluation Laboratory (DEMATEL)

Gabus & Fontela developed DEMATEL in 1973 at the Geneva Research Centre of the Battelle Memorial Institute. This method represents factors as interrelationships among criteria. Hence, DEMATEL is a complete method for building a structural model involving associations of complex factors. It organizes relationships between the elements within a system using numerical representations of the power of influence [24]. This method has been applied successfully in a diversity of situations, such as developing marketing strategies, developing control systems, solving safety problems, and group decision making [23].

### 3.7. Grey Relational Analysis (GRA)

The grey system theory proposed by Deng (1982) has been widely applied in many fields. The term “grey” interpreted as a color is intended to suggest the amount of known information in control theory. GRA, derived from grey system theory, is particularly useful when dealing with poor, incomplete, and uncertain information. It is suitable for solving problems with complex interrelationships between factors and variables, and it has been successfully applied to solve a variety of MADM problems. The main advantages of GRA are the fact that the results are based on original data and that calculation is straightforward and simple [25].

### 3.8. VIKOR

VIKOR, also known as the compromise ranking method, is an effective tool in multicriteria decision making. The acronym is derived from the word ViseKriterijumska Optimizacija I Kompromisno Resenje. Its multicriteria ranking index is based on a measure of “closeness” to the “ideal” solution. This method was introduced by Opricovic in 2004 for optimization and compromise evaluation in dynamic and complex processes.

VIKOR employs linear normalization, but the normalized values do not depend upon the evaluation unit of a criterion. An aggregating function balances the distance from the ideal solution between individual and ideal satisfaction [22]. It is an efficient MCDA technique for ranking, sorting, and then selecting from a set of conflicting alternatives [26].

### 3.9. Fuzzy

In 1965, Zadeh proposed fuzzy set theory, which has been extensively applied to model the ambiguities of human judgment. It also effectively resolves uncertainties in available information for multiple criteria decision making. A fuzzy MCDA model is used to evaluate selected alternative criteria by using decision pools. The suitability of replacements versus criteria and the significance weights of criteria are evaluated in terms of linguistic values represented by fuzzy numbers [27]. In the fuzzy set, linguistic variables are used to describe fuzzy terms that are then used to map the linguistic variables to numerical variables. The truth values of Boolean logic are replaced with unit intervals in the decision-making process [26].

### 3.10. Goal Programming

In 1955, Charnes first employed goal programming as an MODM tool. Goal programming is an extension of linear programming used to solve problems containing multiple, usually conflicting, objects. It is an optimization procedure for handling multiple conflicting objective measures. It is widely used in multicriteria decision making to combine the logic of optimization with mathematical programming in order to make decisions fulfilling several objectives [28].

### 3.11. Data Envelopment Analysis (DEA)

Data envelopment analysis (DEA) is a mathematical programming technique used for evaluating the competence of an observation relative to a set of similar observations [29]. DEA concentrates on measuring the efficiency of multiple decision-making units in an environment with multiple inputs and outputs. Charnes first introduced DEA in 1978 for performance measurement and then later presented a comprehensive theoretical framework. DEA is a nonparametric method for operations research. There have been several applications of DEA in different industries to measure the impact of a multicriteria decision-making system [21]. To aid the understanding of the general reader, several applied techniques, definitions, and aspects are presented in Table 1.

## 4. Cloud Service Selection Based on MCDA

MCDA is a well-established area within the field of operations research, and it has proven its effectiveness in addressing different complex real-world decision-making problems. In [20], a comparative study is presented involving an infrastructure-as-a-service cloud, using MCDA techniques to select the best service based on performance measurements made by a third party monitoring service against five different criteria. Two prominent classes of MCDA are used, multiattribute utility theory (MAUT) and Outranking. Based upon several types, methods, and categories, we derived a taxonomy for multioptions and multicriteria decision making. The results show that MCDA techniques are indeed effective and can be used for cloud service selection but that different techniques do not select the same service. Hence, more work is needed to identify the most effective MCDA method for cloud selection using an extended data set and much broader criteria. However, these results do reveal that TOPSIS and both outranking methods (ELECTRE and PROMETHEE) are more suitable for this purpose. If the number of available services is very large, then TOPSIS is appropriate because of its computational simplicity. The outranking methods are better in scenarios with a small number of alternatives but a large number of criteria.

### 4.1. Service Selection by AHP

AHP was proposed in order to solve this problem by using a hierarchical structure and systematization [30]. In AHP, a ratio is assigned to each pairwise comparison between issues for each criterion in a hierarchy and also between the criteria themselves. Pairwise comparison results are then organized into a hierarchy with a weight assigned to each criterion, providing both qualitative and quantitative measures.

AHP is used by decision makers in order to make more informed decisions regarding their investment in various technologies. AHP is a multiobjective, multicriteria decision-making approach, employing pairwise comparison to derive a range of preferences from a set of alternatives. This is achieved by determining the responsiveness of the selection process to rapidly changing business rules and criteria. AHP transforms the decision-making process from a subjective judgment into an objective determination. A sophisticated formal mathematical decision model supporting the selection of cloud computing services in a multisourcing scenario is presented in [31]. To select appropriate cloud computing services offered by different providers, these authors consider cost and risk factors in the decision-making process. By employing AHP, the risks are considered when implementing the model in a sustainable comprehensive decision-making approach. This method was validated by a simulation study and by considering realistic scenarios.

A model for applying AHP to task-oriented resource allocation in a cloud computing environment is proposed in [32]. The tasks are compared pairwise according to network bandwidth, task completion time, task costs, and task reliability and then weighted with AHP. Resources are then allocated accordingly. The reciprocal comparison matrix and the induced bias matrix are used to identify inconsistent elements and to improve the consistency ratio. In [33], SaaS products are offered in a cloud computing environment that uses AHP to prioritize product features based on an expert scoring system. In [34], SLA and QoS are applied to distributed resource management in the cloud. AHP enables the system to automatically recognize changes in the dynamic environment, making decisions according to available resources and user requirements.

In [35], a novel AHP-based consumer centered cloud service selection method is introduced that specifically is applied to a medical service cloud environment. This consumer-centered service selection mainly considers the preferences of individual and multiple users. The authors introduce cloud services decision making (CSDM) for making reasonable cloud service selections that are already applicable to other areas for which a similar information model is available [35].

Although AHP is an effective decision-making tool, disadvantages such as decision maker subjectivity can yield uncertainties when determining pairwise comparisons. In [36], fuzzy AHP was proposed to overcome some of these disadvantages. This method allows experts to use fuzzy ratios in place of exact ratios. However, it is often difficult for the expert to exactly quantify his or her opinion of a number within the interval [0, 1]. Therefore, it is more suitable to represent this degree of certainty with an interval [37]. The membership values expressed in this interval value can better fit real-world situations. Therefore, IVFs are suitable to represent the fuzziness of the membership value of their opinions. They combined IVFs with fuzzy AHP to propose a new method to determine the fuzzy weights in interval-valued fuzzy numbers. A fuzzy set can be mathematically expressed by assigning to each possible element in the universe of discourse a value representing its grade of membership in the fuzzy set. In [38], the proposed method has several steps, and IVFs are used to establish the sequence of the computation procedure: first establish the hierarchical structure and obtain expert judgment from the fuzzy weights by using IVF AHP. Then compute the alternative performance rating and integrate it with weight criteria, and finally, rank the alternatives.

Although fuzzy set theory can be applied to decision-making problems possessing a degree of uncertainty, the resulting subjective judgment is always somewhat vague. Fuzzy AHP is applied to compute the fuzzy weights of each criterion based on intervalued fuzzy sets (IVFs). In [38], tuples were employed to express the performance ratings of all variables and to determine the ranking order of possible alternatives according to the preference matrix. In the real world, decision-making problems are fraught with uncertainty. Because the subjective judgments of decision makers are always vague, linguistic variables and interval-valued fuzzy sets are suitable for representing the fuzziness of membership values. Fuzzy AHP was applied to compute the fuzzy weights of each criterion based on interval-valued fuzzy set/linguistic variable tuples to express the performance ratings of all alternatives. The alternatives were then ranked in accordance with the preference matrix. This method can provide the decision maker with greater flexibility in expressing his or her subjective judgment with respect to each alternative [38].

In MCC, one of the aims is to select the optimal cloud path between certain classes of clouds that provide the same service in order to offload particular computation tasks [32]. Because many criteria need to be considered, such as speed, bandwidth, price, security, and availability, a multiple criteria decision analysis approach is required to make correct decisions. Here, AHP is used to determine the weights of the cloud path selection criteria, while fuzzy TOPSIS is used to perform the numerical analysis for ranking.

### 4.2. Service Selection Based on TOPSIS Method

Cloud service selection is a multiple criteria group decision-making (MCDM) problem. The technique for order preference by similarity (OPS) to an ideal solution (TOPSIS) can assist service consumers and providers by analyzing available services using fuzzy opinions. In [39], the authors find that a set of predetermined linguistic variables can be parameterized by triangular fuzzy numbers, which can then be used to evaluate the weights of different criteria and the ratings of alternate web services. They describe a fuzzy TOPSIS numerical example to demonstrate computational efficiency.

Web services are tremendously interactive software components that can be published, located, and invoked practically anywhere on the Web. The increasing number of Web services available raises new challenges related to service discovery, selection, and composition. Machine-readable rich representations of service properties, capabilities, and characteristics can be exploited by reasoning mechanisms to support automated discovery [40]. Present discovery techniques do not consider the user's preferences and expectations. A semiordered preference model is introduced in [41] for content-based service discovery. The aim here is to assist service providers and customers in discovering services that match their expectations and preferences. A fuzzy model for the selection of QoS-aware web services, prioritizing customer preferences, is described in [42].

TOPSIS, described in [42], is a well-known multiple criteria decision-making (MCDM) method. It determines the shortest distance from the positive ideal solution (PIS) and the farthest distance from the negative ideal solution (NIS) in order to select the best alternative. TOPSIS is a popular MCDA technique because of its (1) theoretical rigor, (2) ability to represent the human rationale during selection, and (3) prominence in solving traversal rank. Fuzzy TOPSIS methods are now popular for dealing with imprecise information. In [19], fuzzy TOPSIS was employed to assess the selection of web services. Triangular fuzzy numbers are used to represent linguistic variables as the weights of criteria and as the ratings of web services and can be converted into crisp numbers. This conversion performed using the graded mean integration representation method. The triangular fuzzy number is then used to obtain the PIS and the NIS. Therefore, the fuzzy TOPSIS procedure is efficient, and the computational complexity in the decision-making process is reduced.

In [43], a new user centric service-oriented modeling approach is described, which integrates Fuzzy TOPSIS and service component architecture (SCA). Web service selection and composition are effectively satisfied by a group of service consumers' subjective requirements and preferences in a dynamic environment. This method can translate a group of customers' fuzzy requirements into service requirements and model different levels of hardware and software as services in order to satisfy these requirements. A simulated environment represented by 8∗8 LED matrices on a circuit board, corresponding to an office with different appliances, was used to demonstrate dynamic service selection. Using Fuzzy TOPSIS, the simulated computational efficiency of this system was very high.

### 4.3. Other Methods of Service Selection

A response time-based fuzzy control approach has been proposed for allocation of virtualized resources using a self-tuning fuzzy controller with adaptive output amplification and flexible rule selection. Based on the fuzzy controller, a two-layer QoS provisioning framework, DynaQoS was designed to support adaptive multiobjective resource allocation and service differentiation [44]. The dynamic service placement and replication (DSPR) was designed to manage services in a distributed environment by repetitively searching for the best combination of distributed machines from a resource pool until the working group that best satisfies the requirements of the service is found. DSPR introduces a fuzzy inference engine to perform resource evaluation. The effectiveness of this engine is compared with other existing resource evaluation techniques such as rule-based, constraint-based, and utility-based techniques [45]. For service-oriented architectures (SOAs), a QoS measuring method for enterprise cloud service architecture using ANP was developed through research on enterprise cloud service architecture. The supermatrix is used to calculate the relative superiority of each metric element. QoS-ANP can be extended to rank infinite alternatives based on QoS optimization and can also be used to analyze trade-off decisions with multiattributes [46]. Several applied multicriteria cloud service selection methods are shown in Table 2.

### 4.4. Mixed/Combined Approach

In [43], a new approach for dynamic autonomous resource management and scalability in cloud computing was introduced. In this approach, distributed resource management architecture is adopted for Autonomous Node Agents that are tightly coupled with physical machines in a data center. The PROMETHEE method in particular is employed in MCDA. Simulation results validate this approach's efficiency in terms of scalability, feasibility, and flexibility. It is especially suitable for large data centers compared to centralized approaches, as less migration is needed when applying new configurations. The ease of adding or removing criteria and weights in order to change the configuration makes this a particularly flexible approach.

Evaluating the trustworthiness of a Cloud Service suffers from great uncertainty and complexity. To address this issue, a novel hybrid fuzzy multicriteria group decision-making method, based on a combination of fuzzy set and modified VIKOR methods, was proposed. It addresses various types of conflicting and incommensurable trust criteria and selects appropriate weight-based preferences in order to make a suitable decision [26].

A wide range of criteria is used to assess the quality of cloud computing services. A hybrid fuzzy MCDM approach combining fuzzy DEMATEL, fuzzy DANP, and fuzzy VIKOR has been used to improve service levels and meet user needs in fuzzy environments. This approach solves interdependence and feedback problems in the mobile communications industry and for related value-added service content providers by exploring interrelationships among criteria related to operations [47]. It finds an optimal cloud path selection in an optimal cloud from among a certain class of clouds that provide the same service for offloading the computation tasks. AHP and the TOPSIS in a fuzzy environment have been proposed to decide which cloud is the most suitable for offloading. Hence, AHP is employed to determine the weights for the criteria during cloud-path selection, and fuzzy TOPSIS is used for ranking the cloud services. A numerical analysis is performed to evaluate the model [32].

The selection of cloud service providers is a multicriteria decision-making (MCDM) problem. The cloud service providers with the best technology are not always suitable for a given enterprise. VIKOR is often used to solve these dilemmas. Further complicating matters, incorporating expert opinion always introduces subjectivity and vagueness to the decision-making process. Experts can use linguistic variables to express their opinions, and these variables can be used to define interval-valued fuzzy sets. A decision analysis model combining interval-valued fuzzy sets and VIKOR (IVFVIKOR) is described in [48] that evaluates and selects the suitable cloud service provider. A numerical example is used to illustrate the method's computational processing.

## 5. Conclusion

In this study, we focus on the service selection for cloud computing in multicriteria decision-making situations. We describe the MCDA types and characteristics and present a taxonomic categorization. We compare several methods by synthesizing and reviewing the present literature. Several real-world examples with current applications of different methods are provided. Hence, MCDA has a great effect on and importance in multicriteria decision-making scenarios. We thus summarize several of the advantages and disadvantages, and we present several applications of these MCDA methods in the selection of cloud services. In addition, multicriteria applied methods are summarized and compiled in a comprehensive manner that can be applicable in other research fields. Moreover, different MCDA methods and their unique features are presented and compared, which will aid new researchers in selecting research directions. We envision that this study could be extended for intercloud service selections and for mobile cloud computing.

## Figures and Tables

**Table 1:** Summary of MCDA techniques and capabilities.

Name	Objective	Criteria/approach	Author and year
Goal programming	Application of linear programming to solve problems relating to multiple and conflicting objects	Combination of the logic of optimization with mathematical programming	Charnes et al. (1955)

Fuzzy	Evaluation of significance weights in terms of linguistic values represented by fuzzy numbers	Linguistic variables used to describe fuzzy terms that are then mapped to numerical variables	Zadeh (1965)

DEMATEL	Construction of a structural model involving associations of complex factors	Numerical contextual relations among the elements representing the power of influence	Gabus and Fontela (1973)

DEA	Evaluation of the competence of an observation relative to a set of similar observations	Mathematical programming	Charnes (1978)

AHP	Pairwise comparison of attributes structured in a hierarchal relationship	Useful technique for hierarchical relationship criteria	Thomas L. Saaty (1980)

PROMETHEE	Similar to ELECTRE but differing in the pairwise comparison stage	Considers the degree to which one alternative differs from another	Brans and Vincke (1980)

TOPSIS	Selection of an alternative simultaneously the closest to the ideal solution and the farthest from the anti-ideal solution	Close to ideal but the farthest from anti-ideal	Hwang and Yoon (1981)

GRA	Solution of problems with complex interrelationships between factors and variables	Based on grey system theory	Deng (1982)

ELECTRE	Pairwise comparison among alternatives used to identify and eliminate alternatives dominated by other alternatives	Checks only whether one alternative is better or worse than the other	Roy (1991)

ANP	More general representation of interrelationships among decision levels and attributes	Unidirectional relationships with dependence and feedback instead of hierarchy	Thomas L. Saaty (1996)

VIKOR	Ranking of compromises representing indices derived from a measure of “closeness” to the “ideal” solution	Employs linear normalization	Opricovic (2004)

**Table 2:** Summary of different applied multicriteria methods for cloud service selection.

MCDA technique	Aspects	Attributes	Reference
AHP	Consumer-centered service selection, especially for medical services	User preference	[35]

TOPSIS	QoS-based multiple service selection with fuzzy options	Linguistic variable triangular fuzzy numbers	[39]

PROMETHEE	Dynamic autonomous resource, management, and scalability	Suitable for large data centers	[49]

AHP	Fuzzy AHP with IVFs	2-tuple linguistic variables	[38]

Fuzzy	Fuzzy logic-based resource evaluation technique for the DSPR framework	Fuzzy inference engine for resource evaluation.	[45]

AHP	Identifying the scalability gain of enhanced agility in the selection process	Pairwise comparison	[50]

Fuzzy	Response time-based fuzzy control for the allocation of virtualized cloud resources	Adaptive output amplification and flexible rule selection	[44]

Fuzzy TOPSIS	New user centric service-oriented modeling approach in SCA.	Computational efficiency	[43]

AHP	Decision model to support cloud computing services	Costs and risk factors	[31]

Fuzzy DNAP and fuzzy VIKOR	Exploring interrelationships among criteria related to operations	Solves interdependence and feedback problems.	[48]

AHP and fuzzy TOPSIS	Optimal cloud path among class of clouds to perform offloaded computation tasks	Speed, bandwidth, price, security, and availability	[32]

AHP	Distributed resource management	Considers SLA and QoS	[34]

ANP	QoS measuring method for cloud service architecture	A supermatrix is employed for calculation	[46]

Fuzzy VIKOR	Assesses cloud service trustworthiness using a hybrid model	Weight-based preferences	[26]

IVF and VIKOR	Decision analysis model for service selection	Linguistic variables	[47]

AHP	Task-oriented resource allocation	Bandwidth, task costs, and time	[51]

## Abbreviations

AHP: Analytic hierarchy process
ANP: Analytic network process
CC: Cloud computing
CWS: Component web services
DEA: Data envelopment analysis
DEMATEL: Decision-making trial and evaluation laboratory
DSPR: Dynamic service placement and replication
ELECTRE: Elimination and choice expressing reality
GA: Genetic algorithm
GRA: Grey relational analysis
IVF: Intervalued fuzzy
IVS: Item-based vector similarity
MAUT: Multiple attribute utility theory
MADM: Multiattribute decision making
MCC: Mobile cloud computing
MCDA: Multicriteria decision analysis
MCDM: Multicriteria decision making
MCMP: Multicriteria mathematical problem
MCSP: Multicriteria selection problem
MODM: MultiObjective decision making
PROMETHEE: Preference ranking organization method of enrichment evaluations
QoS: Quality of service
SLA: Service level agreement
SAW: Simple additive weighting
SOA: Service oriented architecture
SVD: Singular value decomposition
TOPSIS: Technique for order preference by similarity (OPS) to an ideal solution (TOPSIS)
UDDI: Universal discovery description and integration
VIKOR: ViseKriterijumska Optimizacija I Kompromisno Resenje
WS: Web services
WSCT: Web service composition tree.
